# Long-term exposure to ambient ultrafine particles and respiratory disease incidence in in Toronto, Canada: a cohort study

**DOI:** 10.1186/s12940-017-0276-7

**Published:** 2017-06-19

**Authors:** Scott Weichenthal, Li Bai, Marianne Hatzopoulou, Keith Van Ryswyk, Jeffrey C. Kwong, Michael Jerrett, Aaron van Donkelaar, Randall V. Martin, Richard T. Burnett, Hong Lu, Hong Chen

**Affiliations:** 1 Department of Epidemiology, Biostatistics, and Occupational Health and Gerald Bronfman Department of Oncology, McGill University, 1020 Pine Avenue, West, Montreal, QC H3A 1A2 Canada; 2 Air Health Science Division, Health Canada, Ottawa, Canada; 3 Public Health Ontario, Toronto, Canada; 4 Institute for Clinical Evaluative Sciences, Toronto, ON Canada; 5 Department of Civil Engineering, University of Toronto, Toronto, Canada; 6 Department of Family and Community Medicine, University of Toronto, Toronto, ON Canada; 7 School of Public Health, University of California, Los Angeles, CA USA; 8 Dalhousie University, Halifax, Nova Scotia, Canada; 9 Harvard-Smithsonian Center for Astrophysics, Cambridge, MA USA; 10 Population Studies Division, Health Canada, Ottawa, Canada; 11 Dalla Lana School of Public Health, University of Toronto, Toronto, ON Canada

**Keywords:** Ultrafine particles, Cohort study, Asthma, Copd, Lung cancer

## Abstract

**Background:**

Little is known about the long-term health effects of ambient ultrafine particles (<0.1 μm) (UFPs) including their association with respiratory disease incidence. In this study, we examined the relationship between long-term exposure to ambient UFPs and the incidence of lung cancer, adult-onset asthma, and chronic obstructive pulmonary disease (COPD).

**Methods:**

Our study cohort included approximately 1.1 million adults who resided in Toronto, Canada and who were followed for disease incidence between 1996 and 2012. UFP exposures were assigned to residential locations using a land use regression model. Random-effect Cox proportional hazard models were used to estimate hazard ratios (HRs) describing the association between ambient UFPs and respiratory disease incidence adjusting for ambient fine particulate air pollution (PM_2.5_), NO_2_, and other individual/neighbourhood-level covariates.

**Results:**

In total, 74,543 incident cases of COPD, 87,141 cases of asthma, and 12,908 cases of lung cancer were observed during follow-up period. In single pollutant models, each interquartile increase in ambient UFPs was associated with incident COPD (HR = 1.06, 95% CI: 1.05, 1.09) but not asthma (HR = 1.00, 95% CI: 1.00, 1.01) or lung cancer (HR = 1.00, 95% CI: 0.97, 1.03). Additional adjustment for NO_2_ attenuated the association between UFPs and COPD and the HR was no longer elevated (HR = 1.01, 95% CI: 0.98, 1.03). PM_2.5_ and NO_2_ were each associated with increased incidence of all three outcomes but risk estimates for lung cancer were sensitive to indirect adjustment for smoking and body mass index.

**Conclusions:**

In general, we did not observe clear evidence of positive associations between long-term exposure to ambient UFPs and respiratory disease incidence independent of other air pollutants. Further replication is required as few studies have evaluated these relationships.

**Electronic supplementary material:**

The online version of this article (doi:10.1186/s12940-017-0276-7) contains supplementary material, which is available to authorized users.

## Background

Short-term exposures to ambient ultrafine particles (UFPs) (<0.1 μm) have been associated with acute changes in physiological measures of cardiorespiratory health [1, 2] but little is known about the long-term health effects of these pollutants. Recently, Ostro et al. [3] applied a chemical transport model to estimate UFP exposures (on a 3 × 3 km scale) for women in the California Teachers Study cohort and reported a positive association between ambient UFPs and ischemic heart disease mortality. Moreover, various sources/components of ambient UFPs were independently associated with ischemic heart disease mortality including elemental/organic carbon, metals (copper and iron), and emissions from gasoline and diesel engines [3]. Ambient UFPs were not associated with all-cause mortality or respiratory mortality (including lung cancer). To our knowledge, this is the only cohort study to date to evaluate the long-term health effects of ambient UFPs and studies have yet to evaluate the relationship between UFPs and the incidence of respiratory diseases.

In this study, we examined the association between long-term exposure to ambient UFPs and the incidence of lung cancer, adult-onset asthma, and chronic obstructive pulmonary disease (COPD) in Toronto, Canada. Long-term ambient UFP exposures were assigned to the residential locations of cohort members using a land use regression model developed for Toronto [4]. Our primary aim in conducting this study was to determine if ambient UFPs are independently associated with respiratory disease incidence after adjusting for other air pollutants including fine particulate air pollution (PM_2.5_) and nitrogen dioxide (NO_2_).

## Methods

### Study population

Our study was conducted using the Ontario Population Health and Environment Cohort (ONPHEC), a large population-based cohort in Ontario Canada. Details of the ONPHEC cohort are provided elsewhere [5]. Briefly, this cohort is comprised of all adults in Ontario who, on April 1, 1996 and onwards, were registered with Ontario’s provincial health insurance plan, resided in Ontario for ≥5 years, and were Canadian-born. The cohort was created through record linkages of population-based health administrative databases developed from the Ontario universal healthcare system.

Our analysis included all cohort members who, on April 1, 1996, resided in the city of Toronto, were aged between 30 and 100 years, and were free of the conditions under investigation. Cohort members were followed until December 31, 2012 to determine incident cases of asthma and chronic obstructive pulmonary disease (COPD). For lung cancer, follow-up began on April 1, 2001 to allow for a minimum 5-year time lag in exposure.

### Outcomes

All health data (including comorbidity data for diabetes, congestive heart failure (CHF), hypertension, acute myocardial infarction (AMI) and all cancers) were obtained from databases housed at the Institute for Clinical and Evaluative Sciences (ICES). This study used the following databases: 1) Registered Persons Database (for age, sex, residential history and health insurance eligibility); 2) Ontario COPD Database (for incident COPD); 3) Ontario Asthma Surveillance Information System (for incident asthma); 4) Ontario Cancer Registry (for incident lung cancer); 5) Census at the dissemination area level (for income quintile, census tract-level unemployment rate, education and mean household income); and 6) National Population Health Survey and Canadian Community Health Survey (for smoking habits and BMI used in the indirect adjustment). The International Classification of Diseases, Ninth Revision (ICD-9) codes for COPD and asthma and the third edition of the International Classification of Diseases for Oncology (ICD-O-3) codes for lung cancer are presented in Table 1.

**Table 1:** Diagnostic codes for selected outcomes and comorbidities in this study

	ICD-9/ICD-O-3 codes
Selected outcomes	
Chronic obstructive pulmonary disease (COPD)	491,492, 496
Asthma	493
Lung cancer	C34.0 - C34.9 ^a^
Comorbid health conditions	
Diabetes	250
Hypertension	401–405
Congestive Heart Failure (CHF)	428
Acute myocardial infarction (AMI)	410
All cancer	140–208

^a^Malignant lung tumors (behaviour code 3)

### Air pollution exposure

Estimates of long-term average exposure to ambient UFP and NO_2_ were derived from land use regression models developed for the city of Toronto [4, 6]. UFP exposure estimates were based on measurements collected in 2014 [4, 7] while NO_2_ exposure estimates were based on measurements collected in 2004 [6]. Briefly, the UFP model was based on data collected from a mobile monitoring campaign conducted over three weeks (2 weeks in summer, 1 week in winter) including data from 405 road segments distributed across the city of Toronto. Recent evidence from Amsterdam suggests that short-term monitoring campaigns can be used to develop models to predict past spatial variations in ambient UFPs [8]. The UFP model used in this study included parameters for the natural logarithm of the distance to highways, major roads, the central business district, Pearson international airport, and bus routes as well as land use variables for park land (100-m buffer), open space (100-m buffer), on-street trees (100-m buffer), and length of bus routes (100-m buffer). The model *R* ^2^ value was 0.67; when evaluated on an external sample of 151 data points the *R* ^2^ value was 0.50 and the mean difference between measured and predicted values was −1385 particles/cm^3^ (95% CI: -3754, 982) [4].

The NO_2_ model was based on data collected from 94 sites across Toronto and included variables for the lengths of expressways (200-m buffer) and major roads (50-m buffer), industrial land use (750-m buffer), density of dwellings (2000-m buffer), 24-h traffic counts (500-m buffer), and being downwind of an expressway within 1500 m. The model *R* ^2^ value for the NO_2_ model was 0.69 and bias was estimated to be less than 4% in cross-validation procedures (a cross-validation *R* ^2^ was not reported) [6]. Estimates of long-term average PM_2.5_ concentrations (1998–2011) were derived from satellite observations at a spatial resolution of approximately 1 km × 1 km as previously described [9]. These estimates have been shown to agree closely with ground-level monitoring data across North America (*R* ^2^ = 0.82) [9].

Three-year moving average exposures (based on place of residence) were used for the main analyses.

### Covariates

Individual level covariate data are limited in the ONPHEC cohort. We compiled the following covariates at baseline: age, sex and five comorbidities including diabetes, CHF, hypertension, AMI and all cancers (ICD-9 codes in Table 1). We further created four neighbourhood-level variables using 1996, 2001, and 2006 Canadian Census Dissemination data: 1) the proportion of recent immigrants; 2) the proportion of population aged ≥15 years who had not completed high school; 3) unemployment rate; and 4) mean household income.

### Statistical methods

We estimated hazard ratios (HRs) using Cox proportional hazards models stratified by sex and one-year age groups. Continuous measures of long-term exposures to UFPs, PM_2.5_ and NO_2_ were included in the models as time-dependent variables using a 3-year moving average. For the incidence of lung cancer, a 5-year time lag in the exposure was used (i.e. the most recent 5-years were excluded from the moving average exposure). Follow-up ended if participants died, became ineligible for provincial health insurance (i.e. movement out of province), moved outside of Toronto, or at the end of follow-up (December 31, 2012).

For all three outcomes, we incrementally adjusted for a series of covariates including comorbidities and neighbourhood-level contextual variables. We also evaluated the impact of including a frailty term for neighbourhood (*n* = 140 in the city of Toronto) to account for any unmeasured confounding factors at the neighbourhood level that may be associated with both exposure and outcomes. Finally, we examined multi-pollutant models including UFPs, NO_2_ and PM_2.5_ and investigated potential effect modification by age and sex. In addition, we conducted stratified analyses to evaluate potential effect modification by NO_2_ in associations between particulate air pollutants and respiratory outcomes. All HRs and 95% confidence intervals (CIs) reflect interquartile range (IQR) increases in air pollution concentrations.

A series of sensitivity analyses were conducted to test the robustness of our results. Specifically, UFP concentrations (our primary exposure of interested) were additionally modelled across quintiles of exposure. In addition, we considered mean annual exposures for each pollutant over other time windows including one and two year moving averages; we restricted analyses to participants who had lived at their baseline address for more than 5 years prior to cohort entry; we adjusted for a linear term for time to account for potential changes in the risk of outcomes of interest over time; we adjusted for distance to major roadways; and we indirectly adjusted for potential confounding by smoking and BMI [10]. For lung cancer, we also tested the sensitivity of the results by repeating the analyses after taking into account 0-year (no time lag) and 2-year lags in the exposure estimates. Finally, models for asthma and COPD were additional adjusted for prevalent lung cancer as a surrogate measure of smoking.

Indirect adjustment analyses (to estimate the association between air pollution exposures and adjustment variables for smoking and BMI) made use of data on 3807 subjects in the city of Toronto from the Canadian Community Health Survey from the 2001, 2003, 2005 and 2007 panels who were between 30 and 100 years of age [10]. The hazard ratios between adjustment variables and respiratory disease incidence were directly calculated from the CCHS data because recent systematic reviews of the associations between our selected incidence outcomes and missing risk factors (i.e. smoking and BMI) were not identified. However, we did compare hazard ratios calculated using the CCHS respondents with those reported in previous studies, and found similar results. Specifically, lung cancer incidence was substantially higher in current (HR = 13.67, 95% CI: 3.46–54.03) and former smokers (HR = 4.18, 95% CI 1.15–15.23) in the CCHS respondents compared to never smokers. Similarly, the Women’s Health Initiative Observational Study (WHI-OS) in the US reported that HRs for lung cancer incidence were 13.44 (95% CI: 10.80–16.75) and 4.20 (95% CI: 3.48–5.08) in current and formers smokers, respectively, relative to never smokers [11]. The population characteristics of the CCHS cohort used for indirect adjustment were also similar to our study populations (i.e. 45% male, mean age of 58 years, and hypertension prevalence of 18.2%).

Details of the indirect adjustment procedure have been describe previously [12]. Briefly, motivated by the theory of partitioned regression for linear regression models, this method allows for adjusting the HRs for these risk factors unavailable in the dataset, while simultaneously controlling for all risk factors included in the Cox models (e.g., comorbidities and neighborhood-level covariates). The method requires estimates of the spatial associations between the variables included in the Cox models and the unobserved variables. These estimated relationships were then used to indirectly adjust for smoking (never, former, or current cigarette smoker) and BMI (<25.0, 25.0–29.9, or ≥30 kg/m^2^) for the entire cohort (see Additional file 1: Table S1). The Delta values reported in Table S1 refer to estimated associations between the omitted variables (i.e. smoking and BMI) and the concentrations of UFPs, PM_2.5_, and NO_2_. Negative values indicate inverse relationships between the prevalence of the omitted variables and levels of pollution.

## Results

In total, 74,543 incident cases of COPD (mean follow-up 14.4 years), 87,141 cases of adult-onset asthma (mean follow-up 14.0 years), and 12,908 cases of lung cancer (mean follow-up 14.6 years) were identified over the follow-up period (Table 2). Men and women were present in our cohort in approximately equal proportions and participants had a mean age of approximately 50 years at baseline (Table 2). Approximately 22% of each baseline cohort population moved outside the city of Toronto during the follow-up period. UFP exposures varied substantially across Toronto ranging from less than 10,000/cm^3^ to more than 100,000/cm^3^ (Table 3). Spatial variations in ambient PM_2.5_ and NO_2_ concentrations were less dramatic but still covered the range of exposures typically observed across Canada (Table 3). Estimated UFP exposures were weakly correlated with PM_2.5_ (*r* = −0.26) and NO_2_ (*r* = 0.23); PM_2.5_ and NO_2_ were also weakly correlated (*r* = 0.22). These correlations are consistent with those previously observed in a panel study of personal air pollution exposures in Canada [13]. A scatter plot of NO2 and UFP concentrations is provided in Additional file 1: Figure S1.

**Table 2:** Baseline characteristics of study subjects and their neighborhoods

Characteristics of the cohort	COPD		Asthma		Lung cancer ^a^	
	Total cohort population	Incident cases	Total cohort population	Incident cases	Total cohort population	Incident cases
Number	1,105,258	74,543	1,057,722	87,141	1,039,128	12,908
%	―	6.7	―	8.2	―	1.2
*Individual risk factors at time of entry*						
Age, years (SD)	51.4 (15.3)	62.2 (13.5)	51.8 (15.4)	51.8 (14.7)	50.7 (14.6)	60.0 (11.0)
Men (%)	47	52.5	47.8	40.2	46.9	54.9
Pre-existing comorbidity (%)						
Diabetes	6.8	11.4	6.8	7.3	6.3	8.0
Chronic obstructive pulmonary disease (COPD)	―	―	1.7	4.2	2.0	8.0
Asthma	6	15.2	―	―	6.7	8.5
Acute myocardial infarction (AMI)	0.7	1.5	0.7	0.7	0.6	1.2
Hypertension	20.8	33.8	20.8	23.4	19.9	26.8
Congestive Heart Failure (CHF)	1.7	4.4	1.9	1.9	1.2	1.9
Cancer	3.7	6.6	3.8	3.5	3.0	―
*Area level risk factors from Canadian Census at the census tract level at baseline*						
Percentage of the population 15 years of age and older with less than a high school education	32	34.2	31.9	33.3	32.0	33.8
Percentage of the population 15 years of age and older without employment	10.3	10.9	10.3	10.9	10.3	10.8
Percentage of recent immigrants	11.1	10.6	11	11.5	11.1	10.8
Average household income with all ages (in $1000 CAN) (SD)	62.1 (37.4)	57.9 (34.8)	62.2 (37.6)	58.9 (35.2)	62.0 (37.3)	57.8 (33.6)

*SD* standard deviation

^a^Lung cancer cohort was followed from April 1, 2001 to December 31, 2012

**Table 3:** Descriptive statistics for estimated long-term average air pollution concentrations at baseline

Pollutant	IQR	Mean (SD)	Min	5th	25th	50th	75th	95th	Max
UFPs (count/cm^3^)	10,097	28,473 (9226)	6772	18,184	22,186	26,000	32,320	58,862	109,759
PM_2.5_ (μg/m^3^)	3.2	10.9 (2.1)	3.9	7.2	9.9	10.7	13.1	13.8	14.9
NO_2_ (ppb)	4.1	21.4 (3.5)	9.9	16.4	19.1	21.1	23.2	32.8	48.9

*SD* standard deviation, *IQR* interquartile range

**Fig. 1:**
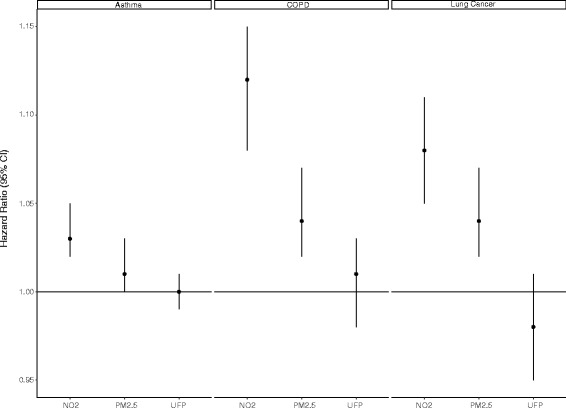
Hazard ratios (HR) and 95% CIs for the incidence of chronic obstructive pulmonary disease (COPD), adult-onset asthma and lung cancer in relation to an IQR increases in each pollutant in Toronto, Canada. All models are stratified by one-year age and sex and are adjusted for neighborhood-level covariates including percentage of the population 15 years of age and older with less than a high school education, percentage of the population 15 years of age and older without employment, percentage of recent immigrants, and average household income. For COPD and asthma, comorbid diabetes, CHF, AMI, hypertension, and all cancers were included as covariates. For lung cancer, we included comorbid diabetes, CHF, AMI and hypertension. Models for UFPs are adjusted for NO_2_ and PM_2.5_. Models for NO_2_ are adjusted for PM_2.5_; PM_2.5_ models are adjusted for NO_2_

Hazard ratios describing the associations between ambient air pollutants and respiratory disease incidence are shown in Table 4. UFP exposures were not associated with increased lung cancer incidence. Small positive associations were observed between UFPs and asthma and COPD in single pollutant models but the magnitudes of these associations decreased when other air pollutants were included in the models (Fig. 1). The relationship between UFPs and COPD was also sensitive (and change directions) to the inclusion of a frailty term for neighbourhood suggesting an important impact of unmeasured confounding factors. When UFP exposures were modelled categorically across quintiles, COPD and asthma incidence were each increased in the four upper quintiles compared to the lowest category of exposure; these risk estimates decreased (but remained elevated) when PM_2.5_ and NO_2_ were included in the models(Table 5).

**Table 4:** Hazard ratios (HR) and 95% CIs for the incidence of chronic obstructive pulmonary disease (COPD), adult-onset asthma and lung cancer in relation to an IQR increases in each pollutant in Toronto, Canada

Exposure	Model	COPD (1996–2012)			Asthma (1996–2012)			Lung cancer ^a^ (2001–2012)		
		HR	95% CI		HR	95% CI		HR	95% CI	
UFPs	UFPs only ^b^	0.96	0.95	0.97	1.02	1.01	1.02	0.97	0.95	0.99
	+ Neighborhood-level covariates ^c^	0.95	0.94	0.96	1.00	0.99	1.01	0.97	0.95	0.99
	+ frailty term for neighborhoods	1.06	1.04	1.08	1.01	1.00	1.02	1.00	0.97	1.03
	+ Medical comorbidities ^d^	1.06	1.04	1.08	1.00	1.00	1.01	1.00	0.97	1.04
	+ PM_2.5_^e^	1.07	1.05	1.09	1.01	1.00	1.02	1.00	0.97	1.04
	+ NO_2_^e^	1.01	0.98	1.03	1.00	0.99	1.01	0.98	0.94	1.01
	+ PM_2.5_ and NO_2_^e^	1.01	0.98	1.03	1.00	0.99	1.01	0.98	0.95	1.01
PM_2.5_	PM_2.5_ only	1.07	1.06	1.09	1.01	1.00	1.02	1.09	1.06	1.12
	+ Neighborhood-level covariates ^d^	1.10	1.08	1.11	1.02	1.01	1.04	1.11	1.07	1.14
	+ frailty term for neighborhoods	1.06	1.04	1.08	1.02	1.00	1.04	1.05	1.03	1.08
	+ Medical comorbidities	1.06	1.04	1.08	1.02	1.00	1.04	1.05	1.03	1.08
	+ NO_2_^e^	1.04	1.02	1.07	1.01	1.00	1.03	1.04	1.02	1.07
NO_2_	NO_2_ only	1.10	1.09	1.11	1.04	1.03	1.05	1.09	1.06	1.11
	+ Neighborhood-level covariates ^d^	1.06	1.05	1.07	1.03	1.02	1.03	1.06	1.04	1.08
	+ frailty term for neighborhoods	1.11	1.07	1.15	1.03	1.02	1.05	1.07	1.04	1.10
	+ Medical comorbidities	1.11	1.07	1.15	1.03	1.02	1.05	1.07	1.04	1.10
	+ PM_2.5_^e^	1.12	1.08	1.15	1.03	1.02	1.05	1.08	1.05	1.11

^a^Cohort for lung cancer was followed up from April 1, 2001 to December 31, 2012 and a 5-year lag in exposure was used

^b^Stratified by one-year age and sex

^c^Neighborhood-level covariates include percentage of the population 15 years of age and older with less than a high school education, percentage of the population 15 years of age and older without employment, percentage of recent immigrants, and average household income

^d^For COPD and asthma, we included comorbid diabetes, CHF, AMI, hypertension and all cancer; For lung cancer, we included comorbid diabetes, CHF, AMI and hypertension

^e^Fully adjusted models

**Table 5:** Hazard ratios (HR) and 95% CIs for the incidence of chronic obstructive pulmonary disease (COPD), adult-onset asthma, and lung cancer across quintiles of ambient UFP concentrations in Toronto, Canada

Exposure	COPD			Asthma			Lung cancer		
	HR	95%CI		HR	95%CI		HR	95%CI	
*Single Pollutant Model*									
UFPs (by quintiles) ^a^									
Q1	1.00			1.00			1.00		
Q2	1.05	1.03	1.09	1.04	1.01	1.06	0.97	0.90	1.03
Q3	1.12	1.08	1.15	1.05	1.02	1.08	1.00	0.92	1.07
Q4	1.16	1.13	1.19	1.06	1.03	1.09	1.03	0.95	1.10
Q5	1.12	1.08	1.17	1.06	1.03	1.10	0.96	0.87	1.05
*Adjusted for NO*_*2*_ *and PM*_*2.5*_									
UFPs ^a^									
Q1	1.00			1.00			1.00		
Q2	1.04	1.02	1.07	1.03	1.01	1.06	0.95	0.89	1.02
Q3	1.08	1.05	1.11	1.04	1.01	1.06	0.97	0.90	1.04
Q4	1.10	1.06	1.13	1.04	1.01	1.07	0.99	0.92	1.07
Q5	1.03	0.98	1.08	1.03	0.99	1.07	0.91	0.82	1.00

^a^Hazard ratios by quintiles of distributions were estimated in the single-pollutant models stratified by age and sex and adjusted for medical comorbidities and neighborhood-level variables. For COPD, quintiles of UFPs: Q1, ≤ 21,473; Q2, 21,473–24,349; Q3, 24,349–27,813; Q4, 27,813–34,763; Q5, > 34,763 count/cm^3^. For asthma, quintiles of UFPs: Q1, ≤ 21,459; Q2, 21,459–24,325; Q3, 24,325–27,788; Q4, 27,788–34,726; Q5, > 34,726 count/cm^3^. For lung cancer, quintiles of UFPs: Q1, ≤ 21,464; Q2, 21,464–24,329; Q3, 24,329–27,793; Q4, 27,793–34,758; Q5, > 34,758 count/cm^3^

Ambient PM_2.5_ and NO_2_ were each associated with modest increases (i.e. 1–12% in fully adjusted models) in the incidence of all three respiratory outcomes and associations were robust to adjustment for medial comorbidities, neighbourhood level covariates, and other air pollutants. However, including a frailty term in models for PM_2.5_ (COPD and lung cancer models) and NO_2_ (COPD model) also impacted risk estimates with hazard ratios increasing for NO_2_ and decreasing for PM_2.5._

For UFPs and NO_2_, associations with incident COPD were strongest among younger subjects; however, other observed associations did not differ substantially by age or sex (Table 6). There was no clear evidence of effect modification by NO_2_ in stratified analyses of associations between particulate air pollutants and respiratory outcomes (Additional file 1: Table S2). Additional adjustment of asthma and COD models for prevalent lung cancer did not materially change results for any of the pollutants (data not shown).

**Table 6:** Hazard ratios and 95% CI for the associations of incident COPD, adult-onset asthma, and lung cancer with an IQR increase in each pollutant by age and sex in Toronto, Canada

Covariates	No. of cases		UFPs			PM_2.5_			NO_2_		
			HR	95% CI		HR	95% CI		HR	95% CI	
*COPD*											
Age	<60	28,854	1.07	1.04	1.11	1.05	1.02	1.08	1.14	1.11	1.17
	60–74	32,092	1.00	0.97	1.03	1.06	1.03	1.08	1.08	1.06	1.11
	> = 75	13,597	1.01	0.98	1.04	1.08	1.05	1.11	1.02	0.99	1.05
Sex	Men	39,111	1.04	1.01	1.06	1.07	1.05	1.10	1.10	1.07	1.14
	Women	35,432	1.06	1.03	1.08	1.06	1.04	1.09	1.10	1.05	1.14
*Asthma*											
Age	<60	58,934	1.01	1.00	1.02	1.01	0.99	1.03	1.04	1.02	1.06
	60–74	21,754	1.00	0.98	1.02	1.04	1.02	1.07	1.03	1.01	1.05
Sex	> = 75	6453	1.01	0.97	1.05	1.02	0.98	1.06	1.00	0.97	1.04
	Men	35,019	1.02	1.00	1.04	1.02	1.00	1.03	1.03	1.01	1.04
	Women	52,122	1.00	0.98	1.01	1.03	1.01	1.04	1.03	1.01	1.05
*Lung Cancer*											
Age	<60	5891	1.00	0.96	1.04	1.04	1.01	1.08	1.04	1.01	1.08
	60–74	5969	1.00	0.97	1.03	1.05	1.03	1.08	1.07	1.04	1.10
Sex	> = 75	1048	1.01	0.93	1.09	1.04	0.98	1.10	1.02	0.93	1.10
	Men	7085	1.00	0.96	1.04	1.06	1.02	1.09	1.09	1.05	1.12
	Women	5823	0.98	0.93	1.03	1.06	1.03	1.10	1.06	1.02	1.10

All models are stratified by age and sex, include a frailty term for neighborhoods, and are adjusted for neighborhood-level covariates

Sensitivity analyses for PM_2.5_ and NO_2_ are shown in Tables 7 and 8. The hazard ratios for PM_2.5_, NO_2_, and lung cancer were sensitive to indirect adjustment for smoking and BMI and the magnitudes of these associations decreased when these parameters were included in the model. The magnitudes of associations between PM_2.5_ and NO_2_ and COPD/asthma were robust (i.e. changes of 1–2%) to various modeling approaches including indirect adjustment for smoking and obesity (Tables 7-8). Hazard ratios for UFPs and lung cancer and asthma remained null in all sensitivity analyses while positive associations were generally observed between UFPs and COPD (Additional file 1: Table S3).

**Table 7:** Sensitivity analyses for the associations of respiratory disease incidence with PM_2.5_

Sensitivity analysis	COPD			Asthma			Lung cancer ^a^		
	HR	95% CI		HR	95% CI		HR	95% CI	
Results from the Main Analyses (Table 4)	1.06	1.04	1.08	1.02	1.00	1.04	1.05	1.03	1.08
Two different time windows of exposure									
1 year before event	1.05	1.04	1.07	1.01	1.00	1.03	NA	NA	NA
2 year before event	1.06	1.04	1.07	1.02	1.00	1.03	NA	NA	NA
Two different lags in the exposure									
0-year lag ^b^	NA	NA	NA	NA	NA	NA	1.04	1.01	1.08
2-year lag ^c^	NA	NA	NA	NA	NA	NA	1.05	1.02	1.08
Restricted to subjects who lived at their baseline addresses for ≥5 years prior to cohort entry	1.06	1.04	1.08	1.02	1.00	1.03	1.05	1.03	1.08
Adjusted for a linear term for time	1.06	1.04	1.08	1.02	1.00	1.04	1.05	1.03	1.08
Adjusted for the distance to roadways	1.06	1.04	1.08	1.02	1.00	1.03	1.05	1.03	1.08
Indirect adjustment (with HRs directly calculated from the CCHS cohort)									
+ Smoking only	1.04	0.99	1.08	1.02	1.00	1.04	1.03	0.95	1.11
+ Smoking and BMI	1.04	0.97	1.09	1.03	1.00	1.05	1.01	0.93	1.10

^a^The cohort was followed up from April 1, 2001 to December 31, 2012

^b^The cohort was followed up from April 1, 1996 to December 31, 2012

^c^The cohort was followed up from April 1, 1998 to December 31, 2012. All models are stratified by age and sex, include a frailty term for neighborhoods, and are adjusted for neighborhood-level covariates

**Table 8:** Sensitivity analyses for the associations of respiratory disease incidence with NO_2_ in fully adjusted models

Sensitivity analysis	COPD			Asthma			Lung cancer ^a^		
	HR	95% CI		HR	95% CI		HR	95% CI	
Results from the Main Analyses (Table 4)	1.11	1.07	1.15	1.03	1.02	1.05	1.07	1.04	1.10
Two different time windows of exposure									
1 year before event	1.11	1.07	1.15	1.05	1.04	1.06	NA	NA	NA
2 year before event	1.11	1.07	1.15	1.05	1.04	1.06	NA	NA	NA
Two different lags in the exposure									
0-year lag ^b^	NA	NA	NA	NA	NA	NA	1.10	1.08	1.13
2-year lag ^c^	NA	NA	NA	NA	NA	NA	1.10	1.07	1.12
Restricted to subjects who lived at their baseline addresses for ≥5 years prior to cohort entry	1.11	1.07	1.15	1.05	1.04	1.06	1.07	1.04	1.10
Adjusted for a linear term for time	1.11	1.07	1.15	1.03	1.02	1.05	1.07	1.04	1.10
Adjusted for the distance to roadways	1.06	1.03	1.09	1.02	1.01	1.03	1.04	1.01	1.07
Indirect adjustment (with HRs directly calculated from the CCHS cohort)									
+ Smoking only	1.08	1.03	1.13	1.03	1.02	1.04	1.02	0.97	1.08
+ Smoking and BMI	1.09	1.04	1.15	1.04	1.02	1.05	1.01	0.95	1.07

^a^The cohort was followed up from April 1, 2001 to December 31, 2012

^b^The cohort was followed up from April 1, 1996 to December 31, 2012

^c^The cohort was followed up from April 1, 1998 to December 31, 2012. All models are stratified by age and sex, include a frailty term for neighborhoods, and are adjusted for neighborhood-level covariates

## Discussion

A number of studies have observed short-term cardiovascular and respiratory health effects of ambient UFPs [1, 2] but little is known about the long-term health effects of these pollutants. We conducted a large population-based study to examine the relationship between long-term exposure to ambient UFPs and respiratory disease incidence in Toronto, Canada. In general, we did not observe clear evidence of positive associations between UFPs and respiratory disease incidence independent of other air pollutants although positive associations were observed between UFPs and incident COPD.

To our knowledge, only one previous cohort study has evaluated the long-term health effects of ambient UFPs. Specifically, Ostro et al. [3] reported positive associations between UFP concentrations (and various components) and ischemic heart disease mortality but UFPs were not associated with mortality from respiratory outcomes. Our findings are consistent with this result as we did not observe clear evidence of associations between ambient UFPs and any of the respiratory outcomes examined. On the other hand, spatial variations in long-term average ambient NO_2_ and PM_2.5_ concentrations were positively associated with lung cancer incidence and this is consistent with existing evidence related to outdoor air pollution and lung cancer risk [14, 15]; however, these associations were sensitive to indirect adjustment for smoking and BMI in our analyses.

In general, it is not clear why UFPs would not be associated with lung cancer incidence as diesel exhaust is a known carcinogen [16] and diesel vehicles are an important source of UFPs in urban areas [7, 17]. One explanation may be that the rapid condensation/agglomeration of UFPs into larger particles tends to concentrate carcinogenic substances into larger particle size fractions and this hypothesis is supported by previous associations between PM_2.5_ and lung cancer [14]. Alternatively, residential estimates of long-term average UFP concentrations may not adequately capture long-term personal exposures as other micro-environments (such as transportation) [7] likely also contribute substantially to UFP exposures. In the future, it may be interesting to incorporate mobility information (e.g. both home and workplace location) into exposure assessment in large population-based studies to see if this has an important impact on effect estimates for pollutants with high spatial variability.

A previous review of epidemiological studies related to air pollution and adult-onset asthma found inconsistent evidence in support of a causal relationship and identified the need for large-scale cohort studies and the inclusion of local-scale traffic pollutants like UFPs [18]. Our investigation addressed both of these needs and our results indicate that NO_2_ and PM_2.5_ may each contribute to increases in the incidence of adult-onset asthma independent of other potential risk factors. The results of two other large cohort studies also support these findings. Specifically, Young et al. [19] reported positive associations between incident adult-onset asthma/wheeze and ambient PM_2.5_ and NO_2_ concentrations among approximately 50,000 women followed over a 4-year period as part of the United States Sister Study cohort. Moreover, a combined analysis of six European cohorts including approximately 24,000 subjects reported positive associations between ambient PM_2.5_/NO_2_ and the development of adult onset asthma [20]. Collectively, evidence from these recent large prospective cohort studies suggests that ambient air pollutants including NO_2_ and PM_2.5_ may contribute to a modest increase in the incidence of adult-onset asthma.

Epidemiological evidence related long-term air pollution exposures and COPD incidence is somewhat limited and remains inconclusive [21]. In particular, two recent cohort studies found little evidence of important relationships between PM_2.5_, NO_2_, and COPD incidence. Specifically, Gan et al. [22] reported weak associations between PM_2.5_, NO_2_, and COPD hospitalizations/mortality in a cohort of approximately 500,000 subjects in Vancouver, Canada followed over a 4-year period. Similarly, a large national cohort study in England including more than 800,000 subjects found little evidence of an important association between PM_2.5,_ NO_2_ and COPD incidence [23]. Conversely, Andersen et al. [24] reported a positive association between long-term exposure to NO_2_ and incident COPD in a Danish cohort study of approximately 57,000 subjects followed between 1993 and 2006. While our findings support modest association between PM_2.5_, NO_2_ and COPD incidence (and possibly UFPs), collective evidence remains limited.

This study had a number of important advantages including large numbers of incident cases and detailed exposure information for multiple air pollutants accounting for subject mobility; however, it is important to note several limitations. First, it is difficult to compare the magnitude of associations across pollutants as different exposure models were used for each air pollutant. Therefore, differences in exposure measurement error might have resulted in more/less bias in associations for a given air pollutant depending on the degree to which residential exposure estimates (assigned to postal code centroids) reflected true long-term personal exposures. This measurement error likely contains components of both Classical (i.e. estimated ambient concentrations distribute around true ambient concentrations) and Berkson type measurement error (true personal exposures distributed around estimated mean ambient concentrations). As this error is likely non-differential with respect to survival time, the Classical measurement error component would tend to bias risk estimates toward the null in proportion to the correlation between measured exposures and true long-term personal exposures (which is unknown and is *not* provided by the model evaluation *R* ^2^ values). The Berkson component of measurement error would not bias risk estimates but would decrease the precision of risk estimates. Therefore, the hazard ratios presented in this study likely underestimate the true magnitudes of associations between long-term exposure to air pollution and respiratory disease incidence.

For UFPs, the exposure model was based on spatial monitoring data collected after the follow-up period and this may have contributed to the null associations observed in our analyses. While analysis of long-term trends would help to clarify this question, the data required to support such an analysis do not exist. Moreover, UFPs are only weakly correlated with ambient NO_2_ and PM_2.5_ and thus trends in these pollutants cannot be used to infer trends in UFPs. What seems likely is that our model may underestimate true long-term exposure levels owing to decreases in vehicle emissions over time. However, spatial *differences* in UFP concentrations are likely more stable over time because spatial differences are primarily impacted by changes in the spatial patterns of diesel traffic (primarily large diesel vehicles). Since large diesel vehicles remain concentrated on major roadways, contrasts between high-traffic and low-traffic areas are likely preserved over time. Therefore, our model likely provides useful estimates of the relationship between health outcomes and *changes* in UFP exposure but may not be appropriate for determining absolute concentrations at which health effects occur (i.e. some minimum exposure threshold).

Another limitation is that we did not have individual level data on smoking behavior which is an important risk factor for all of the outcomes examined. To address this limitation, we used an indirect method of confounder adjustment based on pollutant-outcome associations in a second cohort available in the Toronto area and observed important changes in our effect estimates when this method was applied. However, we cannot rule out residual confounding by smoking.

## Conclusions

We did not observe clear evidence of positive associations between long-term exposure to ambient UFPs and the incidence of lung cancer, COPD, or adult onset asthma. Our findings do suggest a possible association between UFPs and COPD but this association was sensitive to other air pollutants. In general, our findings require further replication in future cohort analyses as few studies have examined the chronic health effects of ambient UFPs to date.

## Additional files

Additional file 1: **Table S1.** Linear associations between smoking and BMI and concentrations of UFPs, PM2.5, and NO_2_. **Table S2.** Hazard ratios (HR) and 95% CIs for the incidence of chronic obstructive pulmonary disease (COPD), adult-onset asthma and lung cancer in relation to an IQR increases in UFPs and PM_2.5_ across tertiles of NO_2_. **Table S3.** Sensitivity analyses for the associations of respiratory disease incidence with UFPs. **Figure S1.** A scatter plot of ultrafine particles (count/cm^3^) and NO_2_ (ppb) concentrations at baseline. (DOCX 77 kb)

## Abbreviations

AMI: Acute myocardial infarction
BMI: Body mass index
CCHS: Canadian community health survey
CHF: Congestive heart failure
COPD: Chronic obstructive pulmonary disease
HR: Hazard ratio
ICD: International classification of diseases
ICES: Institute for clinical and evaluative sciences
ONPHEC: Ontario population health and environment cohort
UFPs: Ultrafine particles
