# Supplementary material for: Long-term exposure to ambient ultrafine particles and respiratory disease incidence in in Toronto, Canada: a cohort study
Source: Environ Health. 2017 Jun 19;16:64. doi: 10.1186/s12940-017-0276-7 (PMC5477122; doi:10.1186/s12940-017-0276-7)

Long-term exposure to ambient ultrafine particles and respiratory disease incidence in in Toronto, Canada: a cohort study

Scott Weichenthal*^1,2^, Li Bai^3,4^, Marianne Hatzopoulou^5^, Keith Van Ryswyk^2^, Jeffrey C. Kwong,^3,4,6^, Michael Jerrett^7^, Aaron van Donkelaar^8^, Randall V. Martin^8 ,9^, Richard T. Burnett^10^, Hong Lu^4^, Hong Chen^3,4,11^

^1^ Department of Epidemiology, Biostatistics, and Occupational Health and Gerald Bronfman Department of Oncology, McGill University, Montreal, Canada

Email: [scott.weichenthal@mcgill.ca](mailto:scott.weichenthal@mcgill.ca)

^2^ Air Health Science Division, Health Canada, Ottawa, Canada

Email: [Keith.VanRyswyk@hc-sc.gc.ca](mailto:Keith.VanRyswyk@hc-sc.gc.ca)

^3^ Public Health Ontario, Toronto, Canada

^4^ Institute for Clinical Evaluative Sciences, Toronto, ON, Canada

Email: [Li.Bai@oahpp.ca](mailto:Li.Bai@oahpp.ca)

^5^ Department of Civil Engineering, University of Toronto, Toronto, Canada
Email: [marianne.hatzopoulou@utoronto.ca](mailto:marianne.hatzopoulou@utoronto.ca)

^6^ Department of Family and Community Medicine, University of Toronto, Toronto, ON, Canada

Email: [jeff.kwong@utoronto.ca](mailto:jeff.kwong@utoronto.ca)

^7^ School of Public Health, University of California, Los Angeles, CA, USA

Email: [mjerrett@ucla.edu](mailto:mjerrett@ucla.edu)

^8^ Dalhousie University, Halifax, Nova Scotia, Canada ^9^ Harvard-Smithsonian Center for Astrophysics, Cambridge, MA, USA

Email: [randall.martin@dal.ca](mailto:randall.martin@dal.ca)

^10^Population Studies Division, Health Canada, Ottawa, Canada

Email: [rick.burnett@hc-sc.gc.ca](mailto:rick.burnett@hc-sc.gc.ca)

^11^ Dalla Lana School of Public Health, University of Toronto, Toronto, ON, Canada

Email: [hong.chen@oahpp.ca](mailto:hong.chen@oahpp.ca)

*Corresponding Author

Dr. Scott Weichenthal, 1020 Pine Ave. West, Montreal, QC, Canada, H3A 1A2

Telephone: 514-398-1584; Email: [scott.weichenthal@mcgill.ca](mailto:scott.weichenthal@mcgill.ca)

Table S1. Linear associations between smoking and BMI and concentrations of UFPs, PM_2.5_, and NO_2_

| Missing risk factors | UFPs | | PM_2.5_ | | NO_2_ | |
| --- | --- | --- | --- | --- | --- | --- |
|  | Delta | SE | Delta | SE | Delta | SE |
| Smoking Status |  |  |  |  |  |  |
| Never smoker (reference category) | - | - | - | - | - | - |
| Current smoker | 0.00169 | 0.00019 | 0.03186 | 0.00360 | 0.02192 | 0.00249 |
| Former smoker | 0.00547 | 0.00183 | 0.00796 | 0.00266 | 0.00676 | 0.02299 |
| BMI (kg/m^2^) |  |  |  |  |  |  |
| <25.0 (reference category) | - | - | - | - | - | - |
| 25.0-29.9 | 0.00338 | 0.00256 | 0.00053 | 0.00045 | 0.00135 | 0.00114 |
| ≥ 30 | 0.00397 | 0.00109 | 0.00895 | 0.00243 | 0.00978 | 0.00260 |

^a^ Adjusted for neighborhood-level covariates and an indicator of neighborhoods from the Canadian Community Health Survey

Table S2. Hazard ratios (HR) and 95% CIs for the incidence of chronic obstructive pulmonary disease (COPD), adult-onset asthma and lung cancer in relation to an IQR increases in UFPs and PM_2.5_ across tertiles of NO_2_.

| Outcome | NO_2_ level |  | UFPs | | | PM_2.5_ | | |  |
| --- | --- | --- | --- | --- | --- | --- | --- | --- | --- |
|  |  | Number of cases | HR | 95%CI | | HR | 95%CI | |  |
| COPD | <Q1 | 23,897 | 1.01 | 0.97 | 1.05 | 1.03 | 1.00 | 1.06 | |
|  | Q1-Q2 | 24,711 | 0.98 | 0.95 | 1.01 | 1.03 | 1.00 | 1.06 | |
|  | >Q2 | 25,935 | 0.98 | 0.95 | 1.01 | 1.03 | 1.00 | 1.06 | |
| Asthma | <Q1 | 28,271 | 1.02 | 0.99 | 1.05 | 1.03 | 1.00 | 1.05 | |
|  | Q1-Q2 | 28,613 | 0.99 | 0.97 | 1.01 | 1.02 | 1.00 | 1.04 | |
|  | >Q2 | 30,257 | 0.99 | 0.97 | 1.01 | 1.00 | 0.97 | 1.02 | |
| Lung cancer | <Q1 | 4,208 | 0.94 | 0.87 | 1.02 | 1.05 | 1.01 | 1.10 | |
|  | Q1-Q2 | 4,302 | 0.94 | 0.88 | 0.99 | 1.06 | 1.01 | 1.10 | |
|  | >Q2 | 4,398 | 0.97 | 0.93 | 1.02 | 1.06 | 1.01 | 1.10 | |

Q1: the 33rd percentile (19.8ppb); Q2: the 66th percentile (22.3ppb). All models are stratified by age and sex, include a frailty term for neighborhoods, and are adjusted for neighborhood-level covariates.

Table S3. Sensitivity analyses for the associations of respiratory disease incidence with UFPs.

| Sensitivity analysis | COPD | | |  | Asthma | | |  | Lung cancer *^a^* | | |
| --- | --- | --- | --- | --- | --- | --- | --- | --- | --- | --- | --- |
|  | HR | 95% CI | |  | HR | 95% CI | |  | HR | 95% CI | |
| Two different time windows of exposure |  |  |  |  |  |  |  |  |  |  |  |
| 1 year before event | 1.08 | 1.06 | 1.10 |  | 1.02 | 1.00 | 1.03 |  | NA | NA | NA |
| 2 year before event | 1.07 | 1.05 | 1.09 |  | 1.02 | 1.00 | 1.03 |  | NA | NA | NA |
| Two different lags in the exposure |  |  |  |  |  |  |  |  |  |  |  |
| 0-year lag *^b^* | NA | NA | NA |  | NA | NA | NA |  | 1.02 | 0.99 | 1.06 |
| 2-year lag *^c^* | NA | NA | NA |  | NA | NA | NA |  | 1.00 | 0.97 | 1.04 |
| Restricted to subjects who lived at their baseline addresses for > 5 years prior to cohort entry | 1.06 | 1.04 | 1.08 |  | 1.01 | 1.00 | 1.03 |  | 1.00 | 0.97 | 1.04 |
| Adjusted for a linear term for time | 1.06 | 1.04 | 1.08 |  | 1.00 | 0.99 | 1.01 |  | 1.00 | 0.97 | 1.03 |
| Adjusted for the distance to roadways | 0.95 | 0.93 | 0.97 |  | 0.99 | 0.98 | 1.00 |  | 0.96 | 0.92 | 0.99 |
| Indirect adjustment (with HRs directly calculated from the CCHS cohort) |  |  |  |  |  |  |  |  |  |  |  |
| + Smoking only | 1.07 | 1.03 | 1.12 |  | 1.01 | 1.00 | 1.03 |  | 1.02 | 0.95 | 1.09 |
| + Smoking and BMI | 1.07 | 1.03 | 1.12 |  | 1.00 | 0.98 | 1.02 |  | 1.02 | 0.95 | 1.10 |

*^a^* The cohort was followed up from April 1, 2001 to December 31, 2012. ***^b^*** The cohort was followed up from April 1, 1996 to December 31, 2012. ***^c^*** The cohort was followed up from April 1, 1998 to December 31, 2012. All models are stratified by age and sex, include a frailty term for neighborhoods, and are adjusted for neighborhood-level covariates.

Figure S1. A scatter plot of ultrafine particles (count/cm^3^) and NO_2_ (ppb) concentrations at baseline.


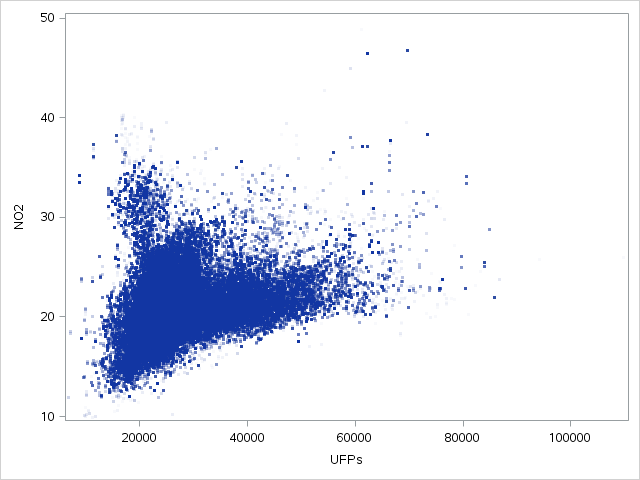

Supplement: Supplementary file 1 — Table S1. Linear associations between smoking and BMI and concentrations of UFPs, PM2.5, and NO2. Table S2. Hazard ratios (HR) and 95% CIs for the incidence of chronic obstructive pulmonary disease (COPD), adult-onset asthma and lung cancer in relation to an IQR increases in UFPs and PM2.5 across tertiles of NO2. Table S3. Sensitivity analyses for the associations of respiratory disease incidence with UFPs. Figure S1. A scatter plot of ultrafine particles (count/cm3) and NO2 (ppb) concentrations at baseline. (DOCX 77 kb) [file 12940_2017_276_MOESM1_ESM.docx]
